# Continuous high and low temperature induced a decrease of photosynthetic activity and changes in the diurnal fluctuations of organic acids in *Opuntia streptacantha*

**DOI:** 10.1371/journal.pone.0186540

**Published:** 2017-10-23

**Authors:** Zaida Zarely Ojeda-Pérez, Juan Francisco Jiménez-Bremont, Pablo Delgado-Sánchez

**Affiliations:** 1 Laboratorio de Biotecnología. Facultad de Agronomía y Veterinaria. Universidad Autónoma de San Luis Potosí. Soledad de Graciano Sánchez, SLP., México; 2 Laboratorio de Cultivo de Tejidos Vegetales. Bioplasma. Escuela de Ciencias Biológicas. Facultad de Ciencias Básicas. Universidad Pedagógica y Tecnológica de Colombia. Sede central. Tunja, Boyacá, Colombia; 3 Laboratorio de Biología Molecular de Hongos y Plantas. División de Biología Molecular. Instituto Potosino de Investigación Científica y Tecnológica, A.C. San Luis Potosí, SLP., México; Instituto de Biologia Molecular y Celular de Plantas, SPAIN

## Abstract

*Opuntia* plants grow naturally in areas where temperatures are extreme and highly variable in the day during the entire year. These plants survive through different adaptations to respond to adverse environmental conditions. Despite this capability, it is unknown how CAM photosynthetic activity and growth in *Opuntia* plantlets is affected by constant heat or cold. Therefore, the main objective of this research was to evaluate the short-term effect of high (40°C) and low (4°C) continuous temperatures on the photosynthetic efficiency, the organic acid content (malic acid) and the relative growth rate (RGR) in seven-month-old *Opuntia streptacantha* plantlets during 5, 10, and 15 days. Chlorophyll fluorescence analysis allowed us to determine that high temperatures negatively impact the photosynthetic efficiency of *O*. *streptacantha* plantlets, which exhibited the lowest values of maximum quantum efficiency of the photosystem II (*F*_*v*_*/F*_*m*_ = 52%, F_v_/F_0_ = 85%), operational quantum yield of PS (ΦPSII = 65%) and relative electron transport rate (rETR = 65%), as well as highest values of basal fluorescence (*F*_*0*_ = 226%) during 15 days of treatment. Similarly, low temperatures decreased *F*_*v*_*/F*_*m*_ (16%), F_v_/F_0_ (50%), Φ_PSII_ and rETR (16%). High temperatures also decreased nocturnal acidification in approximately 34–50%, whereas low temperatures increased it by 30–36%. Additionally, both continuous temperatures affected drastically diurnal consumption of malic acid, which was related to a significant RGR inhibition, where the specific photosynthetic structure area component was the most affected. Our results allowed determining that, despite the high tolerance to extreme temperatures described for *Opuntia* plants, young individuals of *O*. *streptacantha* suffered photosynthetic impairment that led to the inhibition of their growth. Thus, the main findings reported in this study can help to predict the potential impact of climatic change on the establishment and survival of succulent species of arid and semiarid regions of Mexico.

## Introduction

Over the past 100 years, the global average temperature has changed and it is projected to continue changing at a rapid rate [1]. Because of this changes in the temperature patterns, plant communities are exposed to colder winters and hotter summers, which limits the establishment and growth of plants [2].

It is known that temperature affects the main physiological and biochemical processes in plants. The physiological processes most affected are photosynthesis, growth and respiration when temperatures are above or below the normal ranges of growth [3]. In the same way, biochemical changes such as alterations in the viscosity, permeability and fluidity of the cell membrane are produced [3] and, at the enzymatic level, studies have reported changes or inhibition of Ribulose-1,5-bisphosphate carboxylase/oxygenase (RuBisCO), phosphoenol pyruvate carboxylase (PEP-case), pyruvate phosphate dikinase (PPDK), adenosine triphosphate (ATP) synthase, antioxidant enzymes, among others [3,4].

Most plants slow their growth at temperatures above 40°C and under 10°C mainly due to a reduction or inhibition of photosynthesis [5,6]. Nevertheless, xerophyte plants such as *Opuntia* spp. can grow in areas where temperatures at certain times can reach either values over 50°C or below 6°C [7], since they exhibit anatomical and physiological adaptations that allow them to survive [8]. The main physiological adaptation of these plants is the CAM (Crassulacean Acid Metabolism) photosynthesis. This metabolism permits plants to have a high water use efficiency [9], due to stomatal closure during daytime (at high temperature) and stomatal open during nighttime (at low temperature), when the atmospheric CO_2_ is fixed in the form of malic acid, which is stored in the vacuoles until daytime when it is decarboxylated and assimilated into carbohydrates in the Calvin cycle [10]. Nocturnal accumulation of malic acid and its daily variations can be estimated by titratable acidity, which allow to determine changes in CAM activity and carbon assimilation [11], especially when plants are subjected to environments where temperatures are extreme and highly variable during the day.

*Opuntia* plants are exceptional to tolerate high temperatures during the day as well as low temperatures during the night throughout the year. Nevertheless, the degree of tolerance or sensitivity to high/low temperatures differs depending on the species and the air temperature during the natural process of acclimatization in their habitat [12]. There are several reports on the ability of *Opuntia* spp. to withstand extreme temperatures, e.g. *O*. *ficus indica* can tolerate high temperatures of up to 65°C and temperatures as low as −6°C during one hour, suggesting that this species is extremely tolerant to high temperatures but relatively tolerant to low temperatures [12]. In *O*. *robusta*, it was found that this species tolerated −8°C and 61°C treatments during one hour [10]. Cony et al. (2008) [13] found that *O*. *spinulifera* tolerated –10°C during 18 h and that it was not affected when exposed to continuous heat at 52°C during 5 days. In *O*. *streptacantha*, studies evaluating its responses to cold stress are limited. Under low temperature conditions, Wang, et al. (1997) [14] observed that young cladodes of *O*. *streptacantha* accessions from Mexquitic (Mexico) were more sensitive to cold than *O*. *streptacantha* accessions from Texas. In the same way, Valdez-Cepeda et al. (2001) [15] found that mature cladodes of *O*. *streptacantha* plants are more sensitive to low temperatures than *O*. *leucotricha* and *O*. *robusta* plants. They suggest that this sensitivity might be attributed to the site of collection, because the native species *O*. *leucotricha* and *O*. *robusta* were collected at south central region of Mexico, its site of origin, while *O*. *streptacantha* was collected at the north central region of the country. Moreover, studies evaluating the responses of *O*. *streptacantha* to heat are unknown.

Therefore, *Opuntia* species constitute excellent models that could serve to understand the response mechanisms of the CAM plants to high and low temperatures. The acclimatization to such conditions requires the optimization of photosynthetic activity to decarboxylate malate and to avoid photoinhibition [16]. One of the most commonly used techniques in plant physiology to understand the ability of plants to respond to environmental stresses and how these stresses damage the photosynthetic apparatus is chlorophyll fluorescence (Chl fluorescence). The key parameters (F_v_, F_m_, F_0_, F_v_’, F_m_’, F_0_’) and ratios (F_v_/F_m_, F_v_/F_0_, ΔF/F_m_’ or Φ_PSII_) determined in fluorescence analysis provide detailed information on the functionality of the PSII and the photosynthetic apparatus in intact leaves, at a relatively low cost and a fast and non-invasive way [17]. Therefore, differences in photosynthetic efficiency determined from the parameters and ratios mentioned above can be used to detect physiological changes resulting from environmental conditions such as extreme temperatures [18]. For instance, in the obligate CAM *Euphorbia fractiflexa*, temperatures of 40°C in field induced a decrease of *Fv/Fm* and *Φ*_*PSII*_, suggesting that this response is given by the physiological adaptation to tolerate drastic environmental conditions [19]. Moreover, Musil et al. (2009) [20] found that in southern African succulents growing to high temperatures (40–54°C), a decrement of the photosynthetic performance was the principal cause of massive mortalities of endemic desert plants.

In order to extend our understanding of the capacity of CAM plants to respond and survive under conditions of continuous heat and cold stress, the objective of this research was to determine the effects of high (40°C ± 2°) and low (4°C ± 2°) continuous temperatures on some of the most important physiological variables like photosynthetic efficiency, the malic acid content and the relative growth rate (RGR) in young individuals of *O*. *streptacantha* during 5, 10, and 15 days.

## Materials and methods

### Plant material

Mature fruits of *O*. *streptacantha* were purchased in the local market ‘Mexquitic de Carmona’, SLP, Mexico of the farm located at 22°16’N, 101°07’W with 2,020 masl, located South of the Chihuahuan desert. The seeds were sown in trays containing sterile substrate (Kekkilâ Garden, Finland) and watered daily to field capacity. When the seedlings had a cladode of approximately 1 cm of length, they were transplanted individually in plastic pots of 15 cm diameter and 15 cm depth. Plantlets were kept in the greenhouse, and they were watered every four days to field capacity. At six months of age, 297 plantlets were transferred to growth chambers (Intelligent Artificial Climate Incubator, RTOP series, Model RTOP-800D) for acclimatization during one month at 25°C, 102 ± 2 μmol m^-2^ s^-1^ of PPFD, 50% ± 10 of relative humidity and at 16 h/ 8 h light/dark conditions. The temperature, light (simulating conditions of plantlets under shade of nurse plants), and humidity conditions of each of the chambers were checked daily with sensors during the experiment. After this period of acclimatization, independent groups of plantlets (12 ± 1 cm of height) were exposed to high or low temperature treatments to evaluate the photosynthetic efficiency, titratable acidity, as well as RGR and its components: NAR, SPSA, and LWR during each time (5, 10 and 15 days).

### Temperature treatments

The plantlets were exposed to two different treatments of constant day and night temperatures during 5, 10, and 15 days. The treatments were: cold conditions (4°C ± 2°), heat conditions (40°C ± 2°), and control (25°C ± 2°). The experiment was conducted in independent growth chambers with climatic controlled conditions as previously described.

### Chlorophyll fluorescence

Photosystem II activity was measured using a pulse amplitude modulation fluorometer (MINI-PAM-II, Heinz Walz GmbH, 2014, Effeltrich, Germany) according to the recommended procedures in the user manual [21]. The distance clip 60° 2010A accessory was used to position the fiber optics end-piece relative to the sample (cladode). Measurements to determine the minimum fluorescence (*F*_*o*_*)* and maximum fluorescence (*F*_*m*_*)* in 27 plantlets (9 plantlets/treatment) were performed at pre-dawn. To perform the measurement on each of the plantlets, the distance clip 60° 2010A was placed on a flat part of the upper half of the cladode. This part was previously marked for each plantlet (cladode) to ensure that the measurement was always done in the same place during the course of the 15 days stress treatment. At the same time, the leaf clip holder 2035B was used simultaneously to record the PAR, humidity and temperature. Variable fluorescence (*Fv)* in the dark-adapted state was calculated as: *F*_*v*_ = *F*_*m*_*-F*_*0*_ and the maximum quantum efficiency of PSII was calculated using the formula: *F*_*v*_*/F*_*m*_ = (*F*_*m*_*-F*_*0*_)/*F*_*m*_ [22]. Likewise, its more sensitive form *F*_*v*_*/F*_*0*_ was calculated using the formula: *F*_*v*_*/F*_*0*_ = (*F*_*m*_*-F*_*0*_)/*F*_*0*_ [23]. Subsequently, new measurements of chlorophyll fluorescence were made in the light-adapted state (light conditions growth chambers) every 3 h. These data were used to estimate the effective quantum yield of PS II (Φ_PSII_), which was calculated as Φ_PSII =_ *(Fm’*-*F)*/*Fm’* [24], and the electron transport rate (ETR) that it was estimated as rETR = (PAR*Φ_PSII_). This procedure was performed on days 5, 10, and 15. All data were collected and processed in the WinControl-3 software, version 3.23.

### Measurements of the malic acid content

The content of organic acids (malic acid) and their daily fluctuations were estimated on the cladode of 162 seven-months-old *O*. *streptacantha* plantlets (54 plantlets/treatment). Cladode samples were extracted with a cork borer at predawn, about 2 h before the light period started, when the concentration of organic acids is high; and before dusk (about 2 h before the beginning of the dark period) when their concentration decreases due to the typical diurnal fluctuation in CAM photosynthesis plants [16,25]. Each sample was quickly weighed, frozen in liquid nitrogen and then stored at −70°C for further processing. For titration of organic acids, each sample was placed in a mortar, ground directly in 20 ml of 60% ethanol and boiled for 5 min. Then, the extract was completed at a volume of 25 ml with distilled water, cooled to room temperature and titrated with NaOH 0.01 N to pH 7.0. The methods by Zotz and Andrade (1998) [26] and Hernandez-González and Briones-Villareal, 2007 [16] were used to determine the titratable acidity. Diurnal consumption of malic acid (Delta acidity) was calculated according to the equation: (ΔH^+^) = H^+^(predawn)−H^+^_(pre-dusk)_. Here, (Δ) denotes the difference between acid organic content at predawn and pre-dusk.

### Relative growth rate (RGR)

To determine the changes in seedling biomass over time, a new group of 108 plantlets (36 plantlets/treatment) of *O*. *streptacantha* of seven months of age and similar size were selected for the experiments of RGR. The relative growth rate and its components: net assimilation rate (NAR), specific leaf area (SLA) and leaf weight coefficient (LWR) were assessed at the day zero (before being subjected to temperature treatments), as well as the 5^th^, 10^th^, and 15^th^ days. Measurements were made at the following time intervals: data for each time (5, 10, and 15 days) were calculated from the variation between 0 and 5 days, 0 and 10 days and 0 and 15 days. Cladodes and roots were collected, weighed, measured, and photographed. Then, cladodes and roots were dried in an oven at 70°C for 48 h to determine the dry weight. The photosynthetic area (Cladode area) was determined through the image analysis of three photographs per plantlets using the program ImageJ 1.47v [27,28].

The calculations for determining RGR were performed according to Shipley (2002) [29]. The formula to determine RGR was: RGR = (NAR) (g cm^-2^ d^-1^) x (SLA) (cm^2^ g^-1^) x (LWR) (g g^-1^) [29]. NAR, a physiological component, represents an increase in plant total biomass (TB) per total leaf (or photosynthetic) area (TLA) unit and time (T) unit [NAR = (TB_2_-TB_1_)/(T_2_-T_1_)*2/(TLA_1_ + TLA_2_)] [27]. SLA is a morphological component which is determined by leaf dry matter concentration and leaf thickness [29]. In *Opuntia*, a succulent without leaves, the SLA is the specific photosynthetic structure area (SPSA) [27]. LWR measures the allocation of biomass to photosynthetic structures versus other plant parts [27,29].

### Statistical analysis

Data were analyzed and processed whit STATISTICA version 7 software (StatSoft Inc., Tulsa, OK, USA) [30]. The variables *F*_*v*_*/F*_*m*_, *F*_*v*_*/F*_*0*_, F_0_, diurnal consumption of malic acid and RGR and its components were analyzed through one-way ANOVA when categorical variable or factor evaluated was the temperature. The variables Φ_PSII_, rETR and the content of organic acids were analyzed through two-way ANOVA when categorical variable or factor evaluated was the temperature and time in either hours or days. In all cases, the means were compared between treatments independently for each of the evaluated days (5, 10 and 15) using Tukey’s multiple range test at a *P* < 0.05 except for RGR and its components (NAR, SPSA and LWR), which were compared by Fisher test at a *P* < 0.05. All data for each of the variables evaluated were the mean (*n* = 9) ± SE. Linear regression analysis was performed to test correlations between Δacidity of malic acid and RGR and its components. Slopes of the regressions were compared by analysis of variance using slope coefficients and standard errors.

## Results

### Chlorophyll fluorescence parameters

The photosynthesis status of *O*. *streptacantha* exposed to extreme temperatures (40°C and 4°C) was determined through the use of different parameters of chlorophyll fluorescence, as follows: the maximum quantum efficiency of the photosystem II (*F*_*v*_*/F*_*m*_), its more sensitive indicator of changes in the rates of photosynthetic quantum conversion, the (F_v_/F_0_) [23], operational efficiency of the photosystem II (Φ_PSII_), basal or minimum fluorescence (*F*_*0*_), and relative Electron Transfer Rate (r*ETR*) after 5, 10, and 15 days.

**Fig 1:**
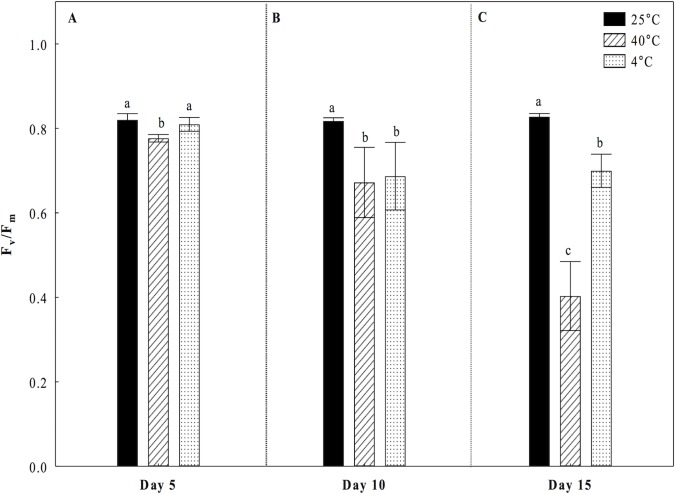
Maximum quantum efficiency of the photosystem II (*F*_*v*_*/F*_*m*_) in cladodes of *Opuntia streptacantha* plantlets. The plantlets were adapted to the dark during overnight and subjected to different temperature treatments for 5 (A), 10 (B), and 15 (C) days. Control (25°C ± 2°), heat (40°C ± 2°), and cold (4°C ± 2°) treatments. The comparison of means between treatments was done independently for each day using Tukey’s multiple range test at a *P* < 0.05. Data are mean (n = 9) ± SE. Different letters denote significant differences between treatments.

**Fig 2:**
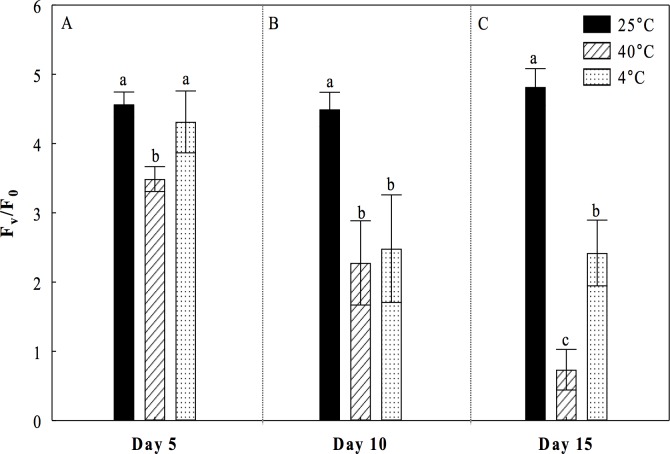
Maximum quantum efficiency of the photosystem II (*F*_*v*_*/F*_*0*_) (more sensitive than *F*_*v*_*/F*_*m*_) in cladodes of *Opuntia streptacantha* plantlets. The plantlets were adapted to the dark during overnight and subjected to different temperature treatments for 5 (A), 10 (B), and 15 (C) days. Control (25°C ± 2°), heat (40°C ± 2°), and cold (4°C ± 2°) treatments. The comparison of means between treatments was done independently for each day using Tukey’s multiple range test at a *P* < 0.05. Data are mean ± SE (n = 9). Different letters denote significant differences between treatments.

**Fig 3:**
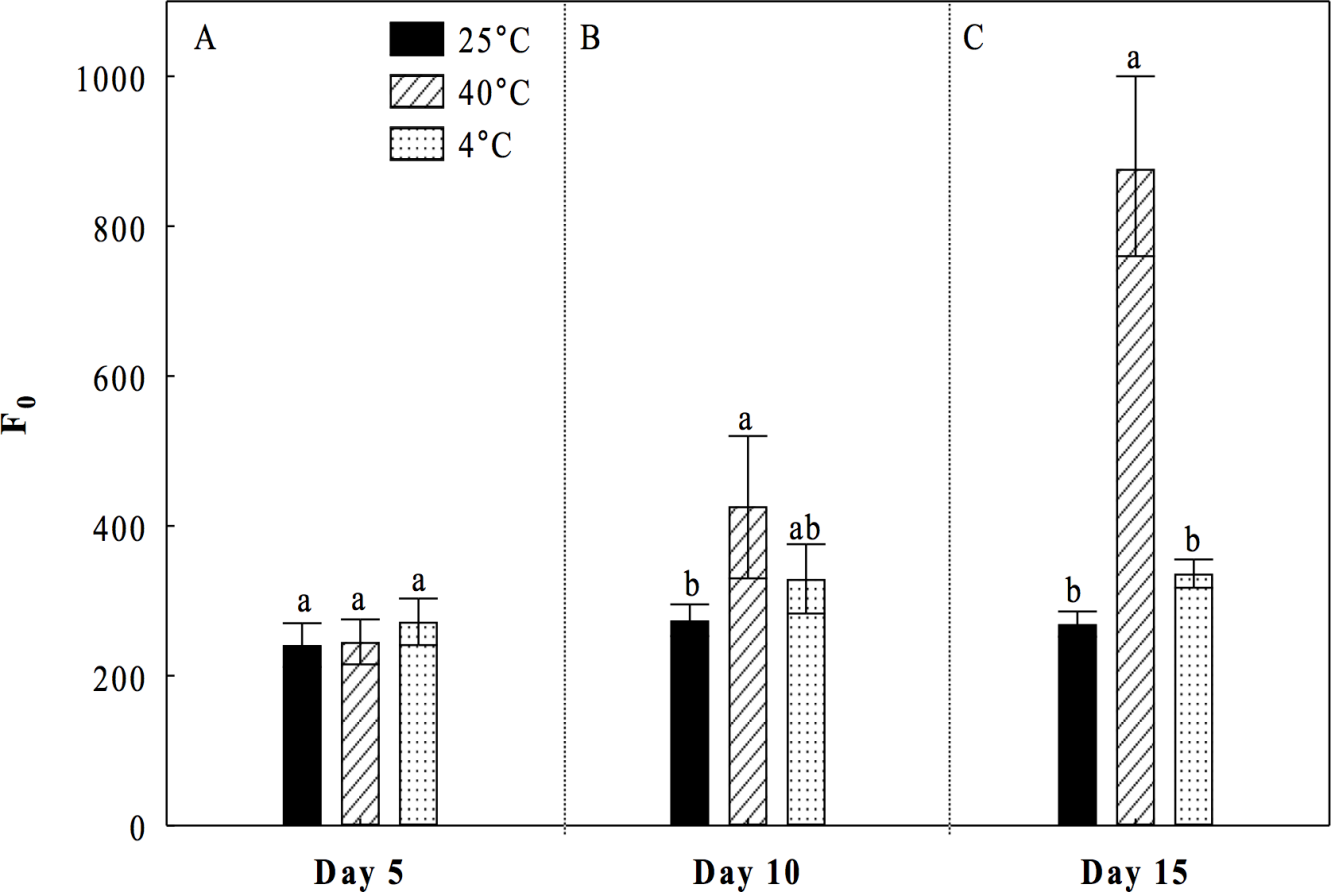
Basal or minimum fluorescence (*F*_*0*_) in cladodes of *Opuntia streptacantha* plantlets. The plantlets were adapted to the dark during overnight and subjected to different temperature treatments for 5 (A), 10 (B), and 15 (C) days. Control (25°C ± 2°), heat (40°C ± 2°), and cold (4°C ± 2°) treatments. The comparison of means between treatments was done independently for each day using Tukey’s multiple range test at a *P* < 0.05. Data are mean ± SE (n = 9). Different letters denote significant differences between treatments.

Under the heat treatment at 40°C, a small but significant decrease of 5.3% in *F*_*v*_*/F*_*m*_ values was observed at day 5 with respect to the control treatment (25°C) (Fig 1A). At the 10^th^ day the decrease in *F*_*v*_*/F*_*m*_ values was greater (18%) (Fig 1B). However, at the 15^th^ day *F*_*v*_*/F*_*m*_ values were significantly lowest than the control treatment with a decrease of 52% (Fig 1C). Under the cold treatment, we observed a significant reduction of 16% in the values of *Fv/Fm* at 10 and 15 days compared to the control treatment (Fig 1B and 1C). The ratio *F*_*v*_*/F*_*0*_ showed very strong declines of 24%, 49% and 85% under the heat treatment, and diminutions of 6%, 45% and 50% under the cold treatment compared to the control treatment during the days 5, 10 and 15, respectively (Fig 2A–2C). The minimum or basal Fluorescence (*F*_*0*_) parameter showed a significant increase after day 5 under heat stress (40°C) respect to the control treatment. In making the assessment of the minimum or basal Fluorescence (*F*_*0*_) parameter, our results showed that by incrementing the time of heat stress (40°C), there is a significant increase in the values of *F*_*0*_ compared to the control, as follows: 56% at the 10^th^ day (Fig 3B), and 226% at the 15^th^ day (Fig 3C). For the treatment of cold conditions at 4°C, no significant differences were observed in F_0_ compared to the control during the days 5, 10, and 15 (Fig 3A–3C).

**Fig 4:**
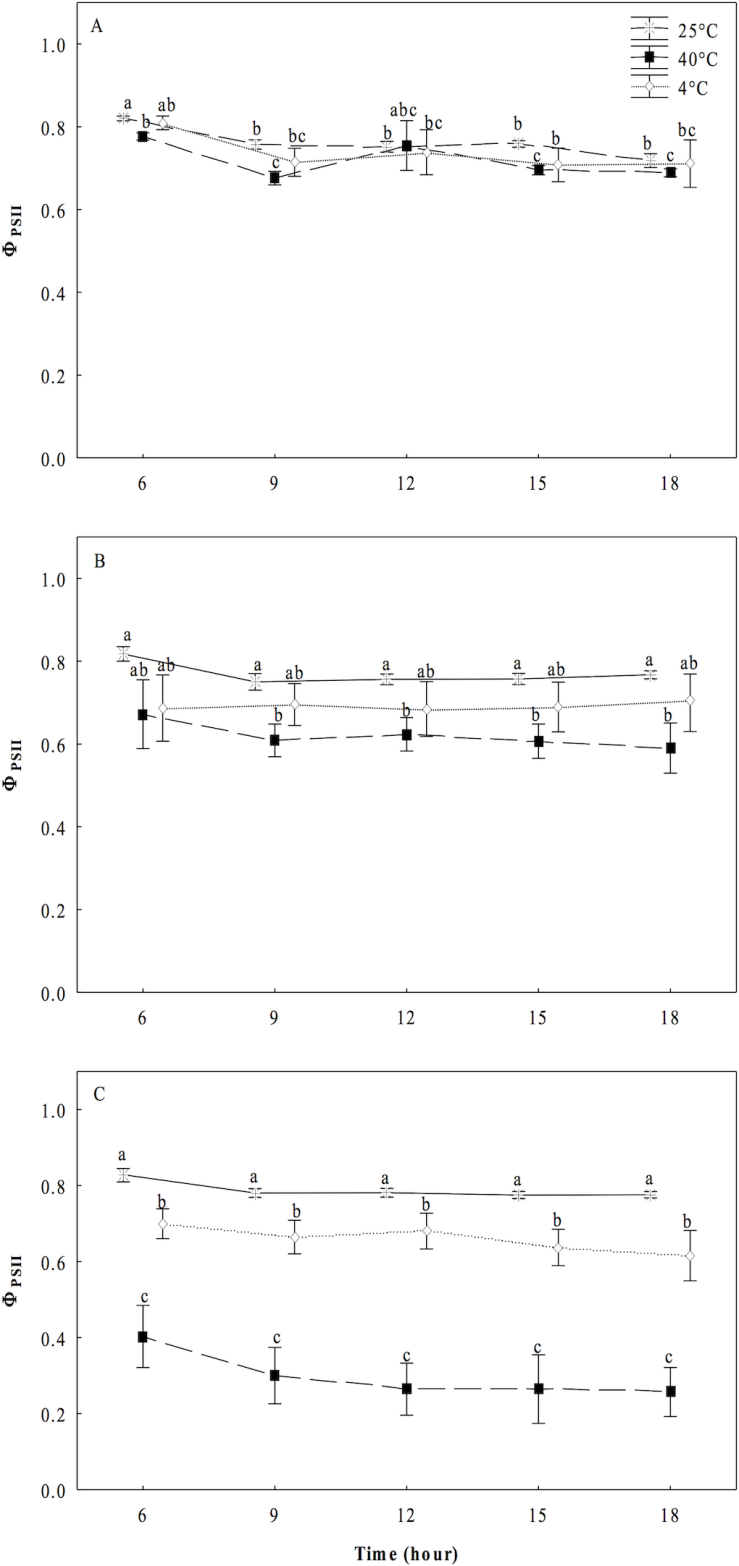
Operational efficiency of the photosystem II (Φ_PSII_) in cladodes of *Opuntia streptacantha* plantlets. Plantlets were subjected to different temperature treatments for 5, 10, and 15 days. Control (25°C ± 2°), heat (40°C ± 2°), and cold (4°C ± 2°) treatments. This parameter was measured every three hours from pre-dawn to pre-dusk. The comparison of means between treatments was done independently for each day using Tukey’s multiple range test at a *P* < 0.05. The comparison of means in each of the days was made within each treatment and between treatments. Vertical bars represent the mean ± SE (*n* = 9).

**Fig 5:**
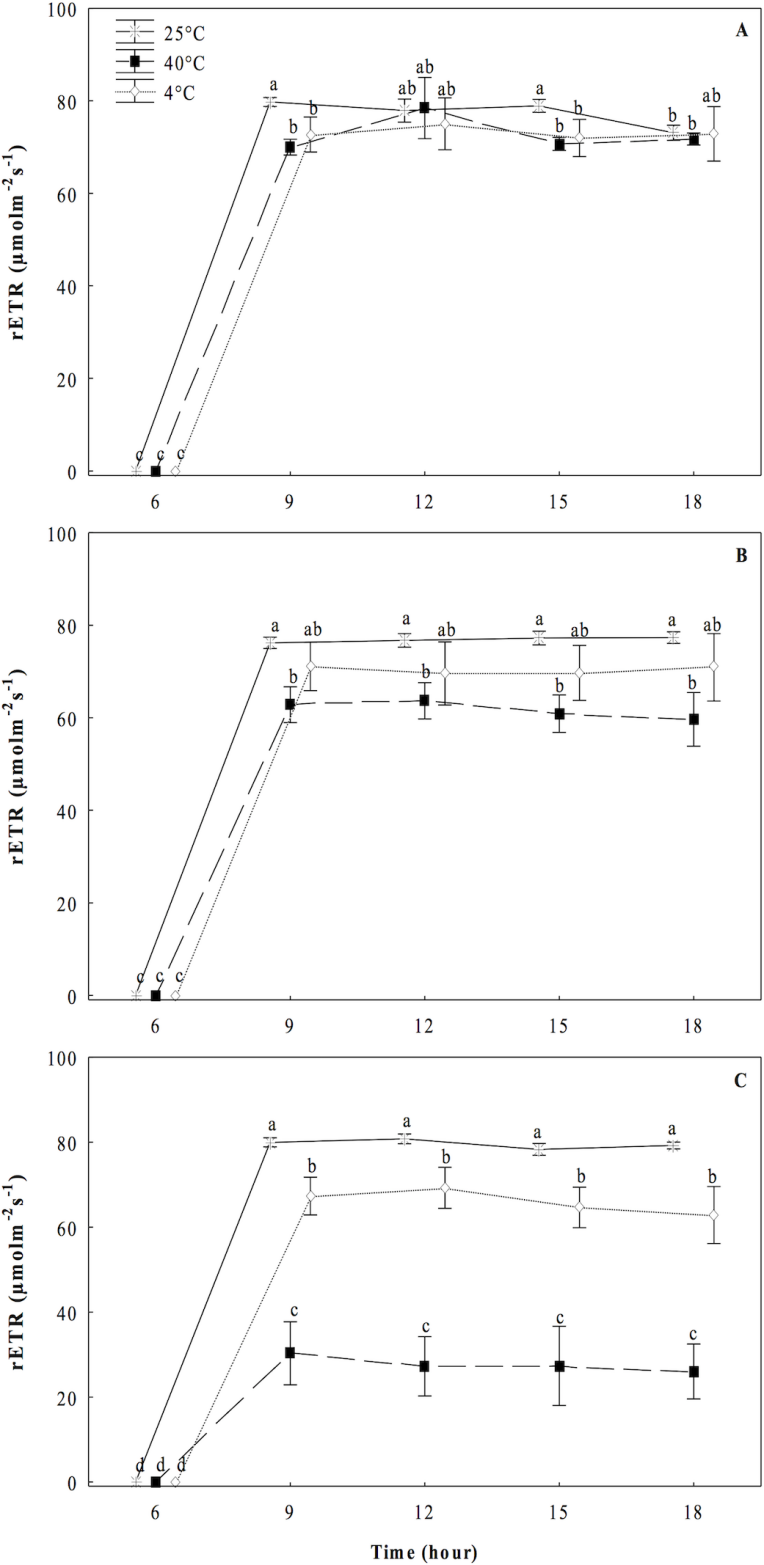
Relative rate of electron flow (r*ETR*) in cladodes of *Opuntia streptacantha* plantlets. Plantlets were subjected to different temperature treatments for 5, 10, and 15 days. Control (25°C ± 2°), heat (40°C ± 2°), and cold (4°C ± 2°) treatments. This parameter was measured every three hours from pre-dawn to pre-dusk. The comparison of means between treatments was done independently for each day using Tukey’s multiple range test at a *P* < 0.05. The comparison of means in each of the days was made within each treatment and between treatments. Vertical bars represent the mean ± SE (*n* = 9).

Finally, we observed a progressive decrement in the values of operational or effective quantum yield of PS II (Φ_PSII_) in *O*. *streptacantha* plantlets under the heat treatment (40°C). The plantlets reduce the Φ_PSII_ to an average of 20% and 65% at 10 and 15 days, respectively (Fig 4A–4C). For the plantlets under the cold treatment (4°C), a slight decrease of an average of 16% was observed at the 15^th^ day compared to the control, presenting statistically significant differences (*P* ≤ 0.05). For rETR values, a trend similar to those of Φ_PSII_ was observed in both high and low temperature treatments (Fig 5A–5C).

### Determination of malic acid content (titratable acidity)

**Fig 6:**
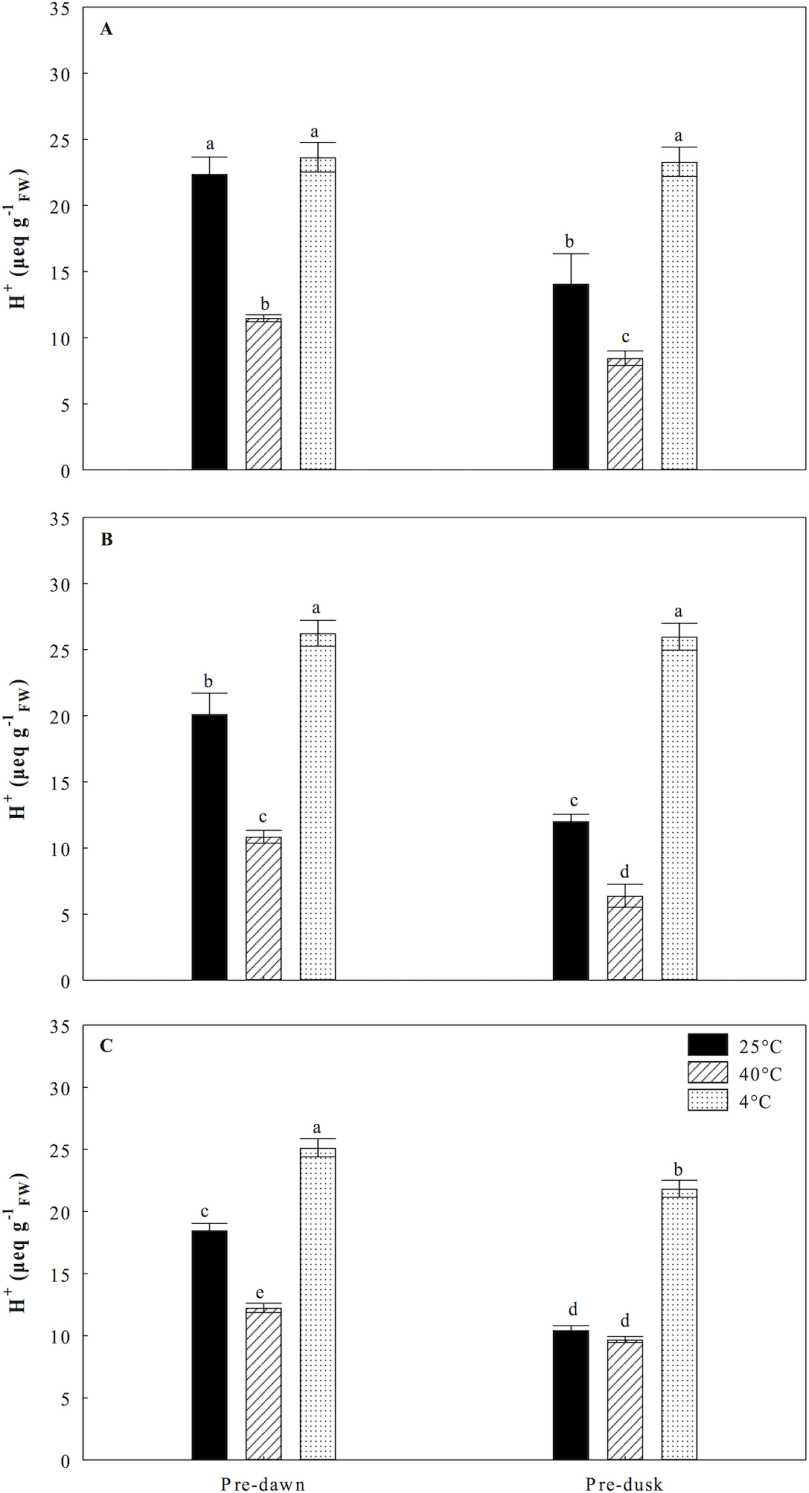
Organic acid content (titratable acidity) of *Opuntia streptacantha* plantlets subjected to extreme temperature treatments during three times. **A**) Day 5; **B)** Day 10, and **C)** Day 15. Control (25°C ± 2°), heat (40°C ± 2°), and cold (4°C ± 2°) treatments. The comparison of means between treatments was done independently for each day using Tukey’s multiple range test at a *P* < 0.05. The comparison of means in each of the days was made within each treatment and between treatments at pre-dawn and pre-dusk. Vertical bars represent the mean ± SE (*n* = 9).

**Fig 7:**
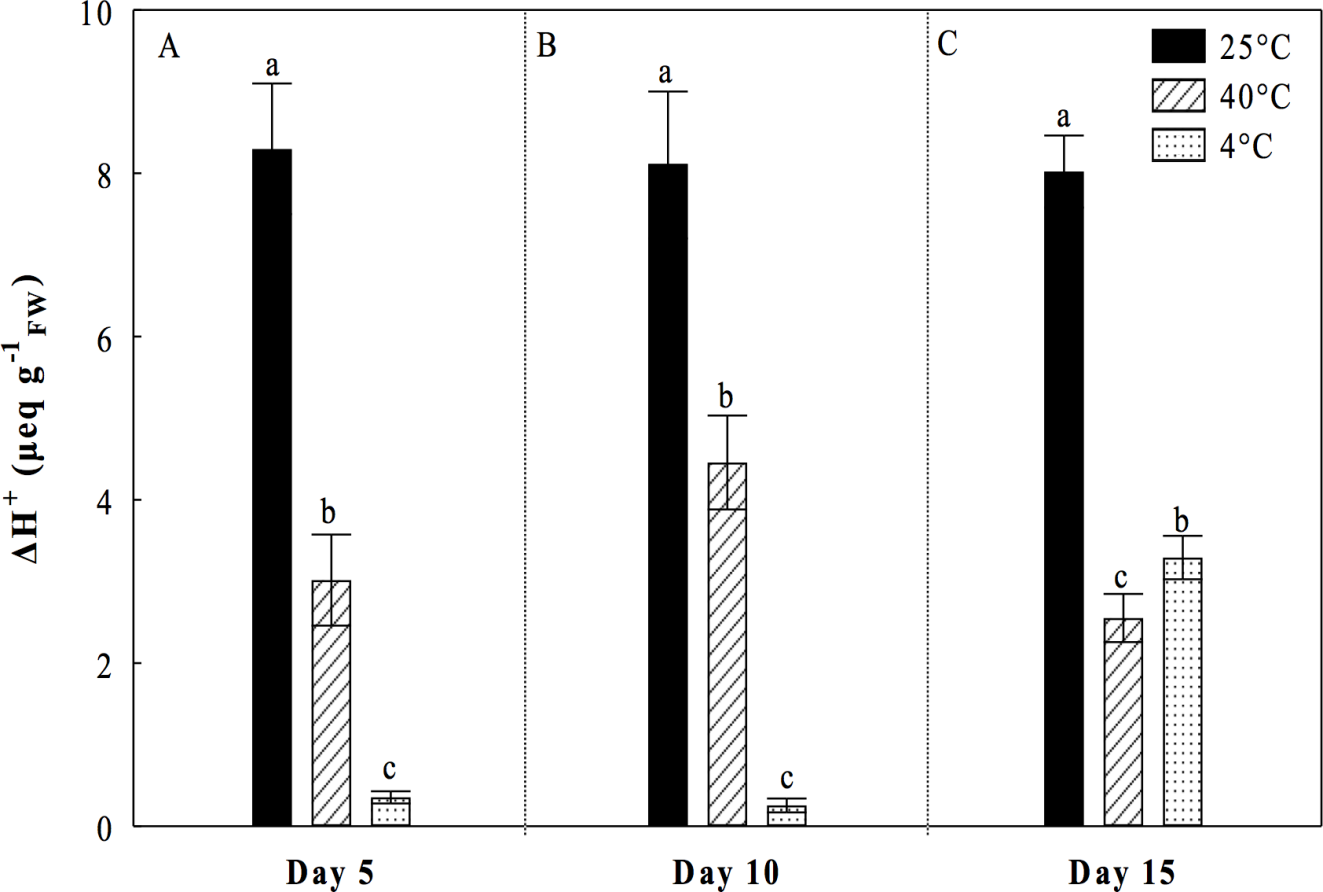
Diurnal consumption of malic acid (ΔH^+^) of *Opuntia streptacantha* plantlets subjected to extreme temperature treatments during three times. **A**) Day 5; **B)** Day 10, and **C)** Day 15. Control (25°C ± 2°), heat (40°C ± 2°), and cold (4°C ± 2°) treatments. The comparison of means between treatments was done independently for each day using Tukey’s multiple range test at a *P* < 0.05. Vertical bars represent the mean ± SE (*n* = 9).

We determined the organic acid content in order to analyze whether the CO_2_ uptake and subsequent diurnal consumption of malic acid could be affected by treatments of continuous temperatures. Under heat stress of 40°C, we found that plantlets of *O*. *streptacantha* showed a significant reduction in nocturnal acidification of 50%, 46% and 31% compared to control at 5, 10, and 15 days, respectively. Despite this reduction the typical CAM fluctuations were observed during the days 5, 10, and 15 (Fig 6A–6C). Nevertheless, significant decreases of 64%, 46% and 69% in diurnal consumption of malic acid were observed respect to the control at 5, 10 and 15 days, respectively (Fig 7A–7C). By contrast, under the cold treatment significant increase in nocturnal acidification of 32% and 44% compared to control on days 10 and 15 were observed (Fig 6B and 6C). Low temperature had a drastic impact on the consumption of malic acid, which was decreased by 96% at the 5^th^ and 10^th^ days (Fig 7A and 7B) and 57% at the 15^th^ day compared to control (Fig 7C).

### Relative growth rate

We analyzed the effect of both high (40°C) and low (4°C) temperature treatments on morphological and physiological variables of RGR. On this regard, a significant inhibition of RGR at 15^th^ day was observed in both treatments respect to the control plants. Nevertheless, a significant decrease of SPSA component was observed at 5, 10 and 15 days for both treatments compared to the control. Low temperatures significantly reduced LWR at the 15^th^ day (Table 1).

**Table 1:** Effect of extreme temperature treatments on RGR and its components of *Opuntia streptacantha* plantlets of seven-month-old.

Treatments	Time (Day)	Relative Growth Rate (RGR)	Net Assimilation Rate (NAR)	Specific Photosynthetic Structure Area (SPSA)	Leaf Weight Ratio (LWR)
25°C	5	0,012 ± 0,002 (a)	0,0009 ± 0,0002 (a)	14,0 ± 0,5 (a)	0,91 ± 0,003 (a)
40°C	5	0,013 ± 0,003 (a)	0,0011 ± 0,0003 (a)	13,0 ± 0,3 (b)	0,91 ± 0,003 (a)
4°C	5	0,008 ± 0,002 (a)	0,0008 ± 0,0002 (a)	11,5 ± 0,2 (c)	0,90 ± 0,003 (a)
25°C	10	0,008 ± 0,002 (a)	0,0006 ± 0,0002 (a)	14,0 ± 0,2 (a)	0,91 ± 0,003 (a)
40°C	10	0,008 ± 0,001 (a)	0,0007 ± 0,0002 (a)	12,0 ± 0,3 (b)	0,91 ± 0,003 (a)
4°C	10	0,007 ± 0,001 (a)	0,0007 ± 0,0001 (a)	11,5 ± 0,2 (b)	0,91 ± 0,002 (a)
25°C	15	0,009 ± 0,001 (a)	0,0007 ± 0,0001 (a)	14,0 ± 0,5 (a)	0,91 ± 0,003 (a)
40°C	15	0,006 ± 0,001 (b)	0,0006 ± 0,0001 (a)	12,0 ± 0,1 (b)	0,91 ± 0,002 (a)
4°C	15	0,006 ± 0,001 (b)	0,0006 ± 0,0001 (a)	11,0 ± 0,2 (c)	0,90 ± 0,004 (b)

Relative growth rate (RGR, g g^-1^ day^-1^), Net assimilation rate (NAR, g cm^-2^ d^-1^), specific leaf area (SLA, cm^2^ g^-1^) and leaf weight coefficient (LWR, g g^-1^). Plants were subjected to different temperature treatments for 5, 10, and 15 days. Control (25°C ± 2°), heat (40°C ± 2°), and cold (4°C ± 2°) treatments. The comparison of means between treatments was done independently for each day using Fisher test at a *P* < 0.05. Data are mean ± SE (n = 9). Different letters denote significant differences between treatments.

### Correlation analysis

Linear regression analysis was performed to test correlations between Δacidity of malic acid and RGR as well as and its components (NAR, SPSA, and LWR). Positive asymptotic relationship between Δacidity of malic acid and SPSA were found for all days of treatment (F = 95,70004, P < 0.001, R^2^ = 0.5477).

## Discussion

Accelerated climate changes over the last 50 years have drastically modified temperature patterns accentuating seasonal variations, so that summers and winters are more intense, especially in arid and semiarid regions [1]. Due to these temperature changes, sessile organisms such as the plants result more affected. As a consequence, their growth and productivity get reduced [3]. Therefore, understanding the ability of plants, especially desert plants, to adjust their physiology to extreme temperatures is of great importance.

In this study, we exposed *Opuntia streptacantha* plantlets to constant temperatures of 40°C or 4°C day/night for 5, 10, and 15 days. Our data indicated that *O*. *streptacantha* plantlets subjected either high or low temperatures treatments exhibited different degrees of damage in CAM photosynthetic activity. The photosynthetic efficiency of PSII was drastically affected, decreasing more rapidly under heat than under cold conditions. In addition, a negative impact on the nocturnal accumulation of acid organic and diurnal consumption of malic acid was observed, clearly indicating a damage of photosynthesis, which resulted in an inhibition of RGR at day 15. Despite this, the plantlets showed 100% of survival during 15 days of continuous exposure to low and high temperatures, which become an interesting feature considering that: first, in natural conditions these plants grow under daily cycles of variable temperature to which they are adapted; however, they do not grow under constant temperatures of 40°C or 4°C during day and night; and second, for other succulent [20,31] and non-succulent plants [32,33] the ability to survive high temperatures during the day and low temperatures during the night, was not observed, despite day / night temperature fluctuations.

Fluorescence chlorophyll analysis allowed us to determine that under the heat treatment, the *O*. *streptacantha* plantlets exhibited the lowest values of *F*_*v*_*/F*_*m*_, *F*_*v*_*/F*_*0*_, *Φ*_*PSII*_ and r*ETR*, as well as the highest values of *F*_*0*_ after 15 days of treatment, indicating a generalized decrease in the photosynthetic efficiency and therefore an impairment of photosynthesis in young individuals of *O*. *streptacantha*. These results suggest that these plants can become very ineffective at using electrons for carbon fixation, leading to membrane overcharge and PSII damage. In this regard, the ratio *F*_*v*_*/F*_*0*_ was a parameter that allowed discriminating in a clearer way the effect of photoinhibition caused by high temperatures on PSII compared to the ratio *F*_*v*_*/F*_*m*_ especially at day 5. That is because *F*_*v*_*/F*_*0*_ is more sensitive than *F*_*v*_*/F*_*m*_ to detect changes of F_v_ and/or F_0_ under stress conditions. Therefore, it is a much better indicator of changes in the rates of photosynthetic quantum conversion than the ratio *F*_*v*_*/F*_*m*_ [23]. These findings are in accordance with the reported by Baker and Rosenqvist (2004) [34], Murchie and Lawson (2013) [18] and Lichtenthaler, et al. (2005) [17], who indicated that low values of *F*_*v*_*/F*_*m*_, *F*_*v*_*/F*_*0*_ and *Φ*_*PSII*_ can be the result of a photoinhibition or low regulation of PSII caused by a severe stress. Likewise, a decrease in the values of *ETR* and increments of *F*_*0*_, might suggest an impairment to transfer electrons through the PSII [34] caused by a probable loss of function of the reaction centers of PSII and a loss of stability of light harvesting complexes [35], which is one of the main key factors that affect the efficiency of photosynthesis [36].

In *O*. *streptacantha*, as in other *Opuntia* species, studies evaluating the response of the photosynthetic apparatus to continuous heat are lacking. However, our results are closely consistent with those found in another Cacti such as *E*. *platyacanthus*, where it was observed that high temperatures also decreased the values of *Fv/Fm*, *Φ*_*PSII*_ and *ETR* without affecting survival [37]. Nevertheless, the evaluated plants were five years old and these grew under simulated warming with high temperature regimes during the day (41.7°C) and low temperature regimes during the night (4.8°C). Similarly, in *Clussia minor*, a decrease in photosynthetic activity of PSII (*Φ*_*PSII*_) was observed when the plants were subjected to drought and high temperature in field experiments. This decrease in *Φ*_*PSII*_ was attributed to an over-energized of PSII during phase III of CAM photosynthesis [38]. Likewise, the same behavior was observed in other CAMs [19] and succulent plants [20]. In no-succulent plants such as wheat and peach, *Fv/Fm* and *ΦPSII* were dramatically decreased at temperatures between 30°C and 45°C, while the *F*_*0*_ values were greatly increased as result of a severe stress in which complex antenna disconnection and severe disintegration of PSII was observed [32,33].

The analysis of our data under the cold treatment revealed that *O*. *streptacantha* plantlets exhibited a significant decrement of the photosynthetic performance after 10 days of treatment suggesting that the reduction in the values of *F*_*v*_*/F*_*m*_, *F*_*v*_*/F*_*0*_, *ΦPSII* and r*ETR* also reflects a damage of the PSII although not as severe as that observed at 40°C. Given that the decrement was not as severe, results suggest that in *O*. *streptacantha* plantlets of seven-month-old the activity of PSII declines more slowly at low temperatures than at high temperatures. This minor degree of damage of the PSII observed at 4°C could be related to an early process of acclimatization to cold, which is a key short-term strategy for survival, as it has been reported for *Nopalea cochenillifera and Opuntia robusta* [10,39]. It has also been described a greater capacity of stabilization of the photosynthetic apparatus, a more efficient regeneration of the PSII in *Colobanthus quitensis* [40], or even photo-protection processes [41]. We also found that *F*_*0*_ values were similar to those of the control treatment. Dias de Azevedo et al. (2011) [35] suggests that when the *F*_*0*_ values are low, the degree of damage of the photosynthetic apparatus may be lower. Then, the ability to maintain the photosynthetic capacity under a condition of thermal stress could be one of the key characteristics that regulate the establishment and distribution of these plants in the arid region such as the south of the Chihuahuan desert.

The photosynthetic activity in succulent plants can be directly related to the CAM activity and its phases, mainly with the nocturnal CO_2_ uptake when there is less evaporative demand. A significant decrement in nocturnal accumulation of malic acid (Fig 6) and diurnal consumption of malic acid (Fig 7) was observed at 40°C in the present study, suggesting a direct impact of heat on the process of nocturnal uptake and fixation of CO_2_ in the form of organic acids and decarboxylation. This could be related to stomatal closure, denaturation of enzymes and loss of water caused by high temperatures [42]. Nobel and Hartsock (1983) [43] suggested that the decrease of organic acids accumulated at night was related to photoinhibition in plants of *Opuntia ficus-indica*. Our results also suggested possible photoinhibition in *O*. *streptacantha* plantlets as was evidenced by the drastic diminutions of *F*_*v*_*/F*_*m*_, *F*_*v*_*/F*_*0*_, *ΦPSII* and r*ETR* observed (Figs 1, 2, 4 and 5). Nobel (1983) [44] reported that high temperatures (44°C) in *O*. *biguellovii* decreased up to 50% the nocturnal accumulation of acids. Besides, in *Agave angustifolia* submitted under different treatments of temperatures, it was reported that above to 35°C the plants showed negative affectations in the CO_2_ uptake and therefore in the accumulation of organic acids [45]. A similar effect was reported for *Aloe vera* when these plants were exposed to temperatures of 35, 40 or 45°C during one hour [46]. In addition, these authors found that *A*. *vera* had a lethal temperature 50 (LT_50_) of 53.2°C, similar to other plants adapted to arid and semi-arid regions, such as *Prosopis chilensis* with a LT_50_ of 53.3°C [47] and *A*. *tequilana* with LT_50_ of 55°C [48]. For *O*. *streptacantha*, Delgado-Sánchez et al. (2013) [27] found that acidity decreased under drought and high light intensity suggesting the acidity is mainly regulated by the plant water status. In this case, high temperatures could negatively affect the CO_2_ fixation due to stomatal closure, which could lead to possible recycling of respiratory CO_2_ [49]. Despite the decrement in nocturnal acidity the typical CAM fluctuations were observed (Fig 6) indicating the use of malic acid as a substrate for the photosynthetic process [26]. Nevertheless, this diurnal consumption of malic acid was considerably diminished compared to the control (Fig 7), suggesting a diminution of CO_2_ available for the Calvin cycle, which limit the export of carbohydrates for growth and reflects the negative effects of high temperature on the CAM cycle, as was evidenced for *Agave angustifolia* [45]. Thus, in young individuals of *O*. *streptacantha* it could be considered that continuous temperature of 40°C is unfavorable for the nocturnal fixation of CO_2_ and to refix CO_2_ in the Calvin cycle. This was reflected in inhibition growth as described below.

Under the cold conditions treatments, we determined a nocturnal accumulation of organic acids (Fig 6) and a diurnal inhibition of the decarboxylation process in *O*. *streptacantha* plantlets until day 10 (Fig 7A and 7B). Szarek et al. (1974) [50] reported that nocturnal temperatures of 15°C and drought in *O*. *basilaris* induced a high production of organic acids where the malic acid accounted for the 85% from the total. Otherwise, in *O*. *humifusa* was reported that the acidification and the rate of CO_2_ assimilation decreased progressively in winter season as stem water content dropped and shoot production ceased. [51]. However, the process of acidification can also occur in succulents at temperatures near 0°C [52]. In the present study, it also was interesting to note that the typical CAM fluctuations did not appear; the diurnal consumption of malic acid was negligible until the 10^th^ day (Fig 7A and 7B). Nevertheless, limited consumption of acid malic was observed at the 15^th^ day (Fig 7C). This pattern was also observed in plants of *O*. *humifusa*, when no CAM was evident and diurnal acid fluctuations were negligible or limited during winter months [51], which may be due to the passive efflux of malate caused by the inhibitory effect of low temperature on the fluidity of the tonoplast [53]. Then, our results suggest that *O*. *streptacantha* plantlets tend to suppress decarboxylation under cold conditions during 15 days [54], which clearly correlated with an inhibition of growth mainly due to the decrease in SPSA, as indicated below.

Plant growth is determined by the relative growth rate (RGR) and their components which determine the increase in biomass per unit of time [55]. Our results demonstrated that both, high and low temperatures inhibited growth in *O*. *streptacantha* plantlets (Table 1), probably as an effect of the decrease in photosynthetic efficiency and the reduction in the consumption of malic acid. Studies evaluating the effect of continuous temperatures on the growth of young individuals of *O*. *streptacantha* are unknown. However, it has been described that under normal conditions the daily net CO_2_ uptake by CAM plants is lower than for C_3_ and C_4_ [56]. Studies of RGR in *O*. *streptacantha* and *O*. *jaliscana* conducted during 90 days in greenhouse and under conditions of high radiation, humidity and fertility showed that the RGR of *O*. *streptacantha* is lower than that of *O*. *jaliscana* [27,57]. The above suggests that these plants tend to be of relatively slow growth. In this sense, conditions of extreme temperatures can affect negatively the growth. Several studies have shown that high temperatures limit growth in plants [58,59]. We also observed a reduction in the components SPSA and LWR of the RGR of *O*. *streptacantha* plantlets. SPSA component explains up to 80% of the RGR variation and is determined in part by the thickness of the photosynthetic structure [29]. In this sense, it has been proposed that the loss of water in the photosynthetic structures might be related to the decrease in the length and thickness of the cladode, which affects the leaf area and therefore the value of SPSA; as is indicated by Loik and Nobel, (1993) [60] for *O*. *fragilis* and by Goldstein and Nobel, (1994) [61] for *O*. *streptacantha*, *O*. *ficus indica* and *O*. *humifusa*. We also found that low temperatures decrease the leaf weight coefficient (LWR), which suggest a reassignment of resources to the root [27,29]. Therefore, the plants decrease the allocation of resources for the production of biomass to photosynthetic structures. Thus, we may suggest that the reduction of the specific leaf area (SPSA) might be due to a loss of water content in the cladode, to the new reallocation of resources and to the reduction of the photosynthetic activity, which finally cause inhibition growth in *O*. *streptacantha* plantlets.

## Conclusion

This study showed that the heat and cold stress had a clear impact on the photosynthetic activity of PSII, the nocturnal accumulation of organic acids and the diurnal consumption of malic acid, indicating an early impairment of photosynthesis and inhibition of growth in *O*. *streptacantha* plantlets. Our results showed that high temperatures stress had a greater impact than low temperatures; the plantlets subjected at 40°C became very ineffective at using electrons for carbon fixation, leading to possible PSII damage that clearly revealed the reduction of malic acid content and the growth inhibition. On the other hand, the *O*. *streptacantha* plantlets had a lower degree of affectation of the photosynthetic activity under low temperatures compared to high temperatures. Even so, at 4°C diurnal acid fluctuations were negligible or limited, which also may have inhibited the growth. Thus, our results allowed determining that despite the high tolerance to extreme temperatures described for *Opuntia* plants, young individuals of *O*. *streptacantha* suffered severe stress. Therefore, the main findings reported in this study can help to predict the potential impact of climate change on the establishment and survival of young individuals of *O*. *streptacantha* and other succulent species of arid and semiarid regions of Mexico, which actually may be essential for determining the distribution of the species and for the subsistence of populations in these regions.

## References

[1] Kennedy J, Morice C, Parker D, Kendon M. Global and regional climate in 2015. Weather. John Wiley & Sons, Ltd; 2016;71: 185–192. doi: 10.1002/wea.2760

[2] Twardosz R, Kossowska-Cezak U, Pełech S. Extremely Cold Winter Months in Europe (1951–2010). Acta Geophys. 2016;0. doi: 10.1515/acgeo-2016-0083

[3] Hasanuzzaman M, Nahar K, Fujita M. Extreme temperature responses, oxidative stress and antioxidant defense in plants. Plants, Abiotic Stress—Plant Responses Appl Agric. 2013; 169–205. doi: 10.5772/54833

[4] Song Y, Chen Q, Ci D, Shao X, Zhang D. Effects of high temperature on photosynthesis and related gene expression in poplar. BMC Plant Biol. 2014;14: 111 doi: 10.1186/1471-2229-14-111 24774695 10.1186/1471-2229-14-111 PMC4036403

[5] Preston JC, Sandve SR. Adaptation to seasonality and the winter freeze. Front Plant Sci. 2013;4: 167 doi: 10.3389/fpls.2013.00167 23761798 10.3389/fpls.2013.00167 PMC3669742

[6] Hasanuzzaman M, Nahar K, Alam MM, Roychowdhury R, Fujita M. Physiological, biochemical, and molecular mechanisms of heat stress tolerance in plants. Int J Mol Sci. 2013;14: 9643–9684. doi: 10.3390/ijms14059643 23644891 10.3390/ijms14059643 PMC3676804

[7] Granados-Sanchez D, Castaneda-Perez AD. El nopal : historia, fisiología, genética e importancia frutícola Mexico: Trillas; 1991.

[8] Nobel P. Ecofisiología de Opuntia ficus-índica In: Mondragón C, Pérez S, editors. Nopal como forraje. Roma: FAO; 2003 pp. 17–24.

[9] Nobel PS. Environmental biology of agaves and cacti New York: Cambridge University Press; 1988.

[10] Zutta BR, Nobel PS, Aramians AM, Sahaghian A. Low- and High-Temperature Tolerance and Acclimation for Chlorenchyma versus Meristem of the Cultivated Cacti Nopalea cochenillifera, Opuntia robusta, and Selenicereus megalanthus. J Bot. 2011;2011: 1–6. doi: 10.1155/2011/347168

[11] Andrade J-L, De La Barrera E, Reyes-Garcia C, Ricalde MF, Vargas-Soto G, Cervera C. El metabolismo ácido de las Crasuláceas: diversidad, fisiología ambiental y productividad. Bol Soc Bot Mex. 2007;81: 37–50.

[12] Nobel PS, De La Barrera E. Tolerances and acclimation to low and high temperatures for cladodes, fruits and roots of a widely cultivated cactus, Opuntia ficus-indica. New Phytol. 2003;157: 271–279. doi: 10.1046/j.1469-8137.2003.00675.x 10.1046/j.1469-8137.2003.00675.x 33873630

[13] Cony MA, Guevara JC, Trione SO, Estevez OR. Response to Freezing and High Temperatures of Detached Cladodes from Opuntia Species ♦. Trial. 2008; 36–48.

[14] Wang X, Felker P, Paterson A. Environmental Influences on Cactus Pear Fruit Yield, Quality and Cold Hardiness and Development of Hybrids with Improved Cold Hardiness. Plant Dis. 1997;29: 48–59.

[15] Valdez-Cepeda RD, Blanco-Macías F, Gallegos-Vázquez C, Salinas-García GE, Vázquez-Alvarado RE. Freezing Tolerance of Opuntia spp. J Prof Assoc Cactus Dev. 2001;4: 105–115.

[16] Hernández-González O, Briones-Villareal O. Crassulacean acid metabolism photosynthesis in columnar cactus seedlings during ontogeny: The effect of light on nocturnal acidity accumulation and chlorophyll fluorescence. Am J Bot. 2007;94: 1344–1351. doi: 10.3732/ajb.94.8.1344 21636502 10.3732/ajb.94.8.1344

[17] Lichtenthaler HK, Buschmann C, Knapp M. How to correctly determine the different chlorophyll fluorescence parameters and the chlorophyll fluorescence decrease ratio RFd of leaves with the PAM fluorometer. Photosynthetica. 2005;43: 379–393. doi: 10.1007/s11099-005-0062-6

[18] Murchie EH, Lawson T. Chlorophyll fluorescence analysis: A guide to good practice and understanding some new applications. J Exp Bot. 2013;64: 3983–3998. doi: 10.1093/jxb/ert208 23913954 10.1093/jxb/ert208

[19] Al-Turki T, Masrahi YS, Sayed OH. Photosynthetic adaptation of Euphorbia fractiflexa (Euphorbiaceae) and survival in arid regions of the Arabian Peninsula. J Plant Interact. Taylor & Francis; 2014;9: 107–111. doi: 10.1080/17429145.2013.774442

[20] Musil CF, Van Heerden PDR, Cilliers CD, Schmiedel U. Mild experimental climate warming induces metabolic impairment and massive mortalities in southern African quartz field succulents. Environ Exp Bot. 2009;66: 79–87. doi: 10.1016/j.envexpbot.2008.11.008

[21] Walz HG. MINI-PAM-II Manual for Touch- screen Operation [Internet]. Primera. Germany: Heinz Walz GMBH; 2014 Available: http://www.walz.com/downloads/overview.html

[22] Kitajima M, Butler W. Quenching of chlorophyll fluorescence and primary photochemistry in chloroplasts by dibromothymoquinone. Biochim Biophys Acta—Bionergetics. 1975;376: 105/115. doi: 10.1016/0005-2728(75)90209-1 10.1016/0005-2728(75)90209-1 1125215

[23] Babani F, Lichtenthaler H. Light-induced and age-dependent development of chloroplasts in etioled Barley leaves as visualized by determinations of photosynthetic pigments, CO2 assimilation rates and different kinds of chlorophyll fluorescence ratios. J Plant Physiol. Urban & Fischer; 1996;148: 555–566. Available: http://cat.inist.fr/?aModele=afficheN&cpsidt=3132348

[24] Genty B, Briantais J, Baker N. The relationship between the quantum yield of photosynthetic electron transport and quenching of chlorophyll fluorescence. Biochim Biophys Acta—Gen Subj. 1989;990: 87–92. doi: 10.1016/S0304-4165(89)80016-9

[25] Kenyon WH, Holaday a S, Black CC. Diurnal changes in metabolite levels and Crassulacean acid metabolism in Kalanchoë daigremontiana leaves. Plant Physiol. 1981;68: 1002–1007. Available: http://www.pubmedcentral.nih.gov/articlerender.fcgi?artid=426034&tool=pmcentrez&rendertype=abstract 16662040 10.1104/pp.68.5.1002 PMC426034

[26] Zotz G, Andrade J-L. Water relations of two co-occurring epiphytic bromeliads. J Plant Physiol. Gustav Fischer Verlag, Jena; 1998;152: 545–554. doi: 10.1016/S0176-1617(98)80276-9

[27] Delgado-Sánchez P, Yáñez-Espinosa L, Jiménez-Bremont JF, Chapa-Vargas L, Flores J. Ecophysiological and anatomical mechanisms behind the nurse effect: Which are more important? A multivariate approach for cactus seedlings. PLoS One. 2013;8 doi: 10.1371/journal.pone.0081513 24312310 10.1371/journal.pone.0081513 PMC3842958

[28] Rasband W. ImageJ 1.47v, U. S. National Institutes of Health, Bethesda, Maryland, USA, http://imagej.nih.gov/ij/, 1997–2016. [Internet]. Bethesda, Maryland, USA.: National Institutes of Health; 1997. p. 2016. Available: http://imagej.nih.gov/ij/

[29] Shipley B. Trade-offs between net assimilation rate and specific leaf area in determining relative growth rate: Relationship with daily irradiance. Funct Ecol. 2002;16: 682–689. doi: 10.1046/j.1365-2435.2002.00672.x

[30] StatSoft. StatSoft Inc., “Statistica”, Data Analysis Software System, version 7. 2004.

[31] Musil CF, Schmiedel U, Midgley GF. Lethal effects of experimental warming approximating a future climate scenario on southern African quartz-field succulents: A pilot study. New Phytol. 2005;165: 539–547. doi: 10.1111/j.1469-8137.2004.01243.x 15720664 10.1111/j.1469-8137.2004.01243.x

[32] Brestic M, Živčák M, Olšovská K, Hauptvogel P. The photosynthetic heat tolerance and acclimation assessed by fast chlorophyll a fluorescence kinetics in 30 wheat genotypes. In: Libiaková G, Olšovská* K, Gajdošová A, Brestič M, editors. Abstracts from International Scientific Conference ESNA. First. Slovak Republic; 2012. p. 62.

[33] Garbin E, Ramm A, Bacarin MA. The chlorophyll a fluorescence as an indicator of the temperature stress in the leaves of Prunus persica. Brazilian J Plant Physiol. 2012;24: 237–246. doi: 10.1590/S1677-04202013005000001

[34] Baker NR, Rosenqvist E. Applications of chlorophyll fluorescence can improve crop production strategies: An examination of future possibilities. J Exp Bot. 2004;55: 1607–1621. doi: 10.1093/jxb/erh196 15258166 10.1093/jxb/erh196

[35] Dias de Azevedo A, Amorim P, Pereira D, Conceiçao A. Fluorescência da clorofila como uma ferramenta possível para seleção de tolerância á salinidade em girassol. Rev Cienc Agron. 2011;42: 893–897. doi: 10.1590/S1806-66902011000400010

[36] Kaňa R, Govindjee. Role of Ions in the Regulation of Light-Harvesting. Front Plant Sci. 2016;7: 1849 doi: 10.3389/fpls.2016.01849 28018387 10.3389/fpls.2016.01849 PMC5160696

[37] Aragón-Gastelum JL, Flores J, Yáñez-Espinosa L, Badano E, Ramírez-Tobías HM, Rodas-Ortíz JP, et al Induced climate change impairs photosynthetic performance in Echinocactus platyacanthus, an especially protected Mexican cactus species. Flora Morphol Distrib Funct Ecol Plants. Elsevier GmbH.; 2014;209: 499–503. doi: 10.1016/j.flora.2014.06.002

[38] de Mattos E, Herzog B, Luttge U. Chlorophyll fluorescence during CAM-phases in Clusia minor L. under drought stress. J Exp Bot. 1999;50: 253–261. doi: 10.1093/jexbot/50.331.253

[39] Nobel PS, Zutta BR. Temperature tolerances for stems and roots of two cultivated cacti, Nopalea cochenillifera and Opuntia robusta: Acclimation, light, and drought. J Arid Environ. 2008;72: 633–642. doi: 10.1016/j.jaridenv.2007.08.005

[40] Bascuñán-Godoy L, Sanhueza C, Cuba M, Zuñiga GE, Corcuera LJ, Bravo L a. Cold-acclimation limits low temperature induced photoinhibition by promoting a higher photochemical quantum yield and a more effective PSII restoration in darkness in the Antarctic rather than the Andean ecotype of Colobanthus quitensis Kunt Bartl (Cariop. BMC Plant Biol. 2012;12: 114 doi: 10.1186/1471-2229-12-114 22827966 10.1186/1471-2229-12-114 PMC3490872

[41] Osmond CB. Compromising Efficiency: the Molecular Ecology of Light-resource Utilization in Plants In: Press MC, Scholes JD, Baker MG, editors. Physiological Plant Ecology. Oxford: Blackwell Science; 1999 pp. 1–25.

[42] Nobel PS, de la Barrera E. High Temperatures and Net CO2 Uptake, Growth, and Stem Damage for the Hemiepiphytic Cactus *Hylocereus undatus*. Biotropica. 2002;34: 225–231. doi: 10.1646/0006-3606(2002)034[0225:HTANCU]2.0.CO;2

[43] Nobel PS, Hartsock TL. Relationships between Photosynthetically Active Radiation, Nocturnal Acid Accumulation, and CO(2) Uptake for a Crassulacean Acid Metabolism Plant, Opuntia ficus-indica. Plant Physiol. 1983;71: 71–75. doi: 10.1104/pp.71.1.71 16662802 10.1104/pp.71.1.71 PMC1065988

[44] Nobel PS. Low and Higth temperature influences on cacti. In: R. Marcelle, H. Clijsters M van P, editor. Effects of Stress on Photosynthesis In: Advances in Agricultural Biotechnology Volume 3. 1983. pp. 165–174. 10.1007/978-94-009-6813-4

[45] Holtum JAM, Winter K. Limited photosynthetic plasticity in the leaf-succulent CAM plant Agave angustifolia grown at different temperatures. Funct Plant Biol. 2014;41: 843–849. doi: 10.1071/FP13284 10.1071/FP13284 32481038

[46] Huerta C, Freire M, Cardemil L. Expression of hsp70, hsp100 and ubiquitin in Aloe barbadensis Miller under direct heat stress and under temperature acclimation conditions. Plant Cell Rep. 2013;32: 293–307. doi: 10.1007/s00299-012-1363-4 23111788 10.1007/s00299-012-1363-4

[47] Ortiz C, Cardemil L. Heat-shock responses in two leguminous plants: a comparative study. J Exp Bot. 2001;52: 1711–1719. doi: 10.1093/jexbot/52.361.1711 11479337

[48] Luján R, LledÍas F, MartÍnez LM, Barreto R, Cassab GI, Nieto-Sotelo J. Small heat-shock proteins and leaf cooling capacity account for the unusual heat tolerance of the central spike leaves in Agave tequilana var. Weber. Plant, Cell Environ. 2009;32: 1791–1803. doi: 10.1111/j.1365-3040.2009.02035.x 19703117 10.1111/j.1365-3040.2009.02035.x

[49] Chen WH, Tseng YC, Liu YC, Chuo CM, Chen PT, Tseng KM, et al Cool-night temperature induces spike emergence and affects photosynthetic efficiency and metabolizable carbohydrate and organic acid pools in Phalaenopsis aphrodite. Plant Cell Rep. 2008;27: 1667–1675. doi: 10.1007/s00299-008-0591-0 18682955 10.1007/s00299-008-0591-0

[50] Szarek SR, Ting IP. Respiration and Gas Exchange in Stem Tissue of Opuntia basilaris. Plant Physiol. 1974;54: 829–834. doi: 10.1104/pp.54.6.829 16658984 10.1104/pp.54.6.829 PMC366617

[51] Koch KE, Kennedy RA. Effects of seasonal changes in the Midwest on Crassulacean Acid Metabolism (CAM) in Opuntia humifusa Raf. Oecologia. 1980;45: 390–395. doi: 10.1007/BF00540212 28309570 10.1007/BF00540212

[52] Medina E, Delgado M. Photosynthesis and night CO2 fixation in Echeveria columbiana v. Poellr. Photosynthetica. 1976;10: 155–163.

[53] Barker D., Adams W. The xanthophyll cycle and energy dissipation in differently oriented faces of the cactus Opuntia macrorhiza. Oecologia. 1997;109: 353–361. doi: 10.1007/s004420050093 28307531 10.1007/s004420050093

[54] Lakso AN, Kliewer WM. The influence of temperature on malic acid metabolism in grape berries. Plant Physiol. 1975;56: 370–372. 16659305 10.1104/pp.56.3.370 PMC541825

[55] Hunt R, Causton DR, Shipley B, Askew AP. A modern tool for classical plant growth analysis. Ann Bot. 2002;90: 485–488. doi: 10.1093/aob/mcf214 12324272 10.1093/aob/mcf214 PMC4240380

[56] Hartsock T, Nobel P. Watering converts a CAM plant to daytime CO2 uptake. Nature. 1976;262: 574–576. Available: http://dx.doi.org/10.1038/262574b0

[57] Romo-Campos R, Flores-Flores JL, Flores J, Alvarez-Fuentes G. Factores abióticos involucrados en la facilitación entre leñosas y suculentas en el altiplano Mexicano. Bot Sci. 2013;91: 319–333.

[58] Dessena L, Mulas M. Influence of temperature on biomass production of clones of Atriplex halimus. Int J Biometeorol. 2016;60: 677–686. doi: 10.1007/s00484-015-1062-2 26353974 10.1007/s00484-015-1062-2

[59] Angert AL. Growth and leaf physiology of monkeyflowers with different altitude ranges. Oecologia. 2006;148: 183–194. doi: 10.1007/s00442-006-0361-z 16468056 10.1007/s00442-006-0361-z

[60] Loik ME, Nobel PS. Exogenous Abscisic Acid Mimics Cold Acclimation for Cacti Differing in Freezing Tolerance. Plant Physiol. 1993;103: 871–876. 12231985 10.1104/pp.103.3.871 PMC159058

[61] Goldstein G, Nobel P s. Water Relations and Low-Temperature Acclimation for Cactus Species Varying in Freezing Tolerance. Plant Physiol. 1994;104: 675–681. doi: 10.1104/pp.104.2.675 12232118 10.1104/pp.104.2.675 PMC159246

